# Noise Reduction for MEMS Gyroscope Signal: A Novel Method Combining ACMP with Adaptive Multiscale SG Filter Based on AMA

**DOI:** 10.3390/s19204382

**Published:** 2019-10-10

**Authors:** Jingjing He, Changku Sun, Peng Wang

**Affiliations:** 1 State Key Lab of Precision Measuring Technology and Instruments, Tianjin University, Weijin Road, Tianjin 300072, China; jingjing_he_tju@foxmail.com (J.H.); sunck@tju.edu.cn (C.S.); 2 Science and Technology on Electro-Optic Control Laboratory, Luoyang Institute of Electro-Optic Equipment, Luoyang 471000, China

**Keywords:** signal denoising, adaptive chirp mode pursuit, mutual information, adaptive multiscale Savitzky–Golay filter

## Abstract

In this paper, a novel hybrid method combining adaptive chirp mode pursuit (ACMP) with an adaptive multiscale Savitzky–Golay filter (AMSGF) based on adaptive moving average (AMA) is proposed for offline denoising micro-electromechanical system (MEMS) gyroscope signal. The denoising scheme includes preliminary denoising and further denoising. At the preliminary denoising stage, the original gyroscope signal is decomposed into signal modes one by one using ACMP with modified stopping criterion based on mutual information. Useful information is extracted while most noise is discarded in the residue at this stage. Then, AMSGF is proposed to further denoise the signal modes. Sample variance based on AMA is used to adjust the window size of AMSGF adaptively. Practical MEMS gyroscope signal denoising results under different motion conditions show the superior performance of the proposed method over empirical mode decomposition (EMD)-based denoising, discrete wavelet threshold denoising, and variational mode decomposition (VMD)-based denoising. Moreover, AMSGF is proven to gain a better denoising effect than some other common smoothing methods.

## 1. Introduction

The micro-electromechanical system (MEMS) gyroscope, featuring compactness, low cost, and low power consumption, is an important device for measuring the angular velocity of a moving object [1]. However, the accuracy of the MEMS gyroscope quickly degrades over time because of high-level noise emerging from the gyroscope outputs. Therefore, compensating the random drift of the MEMS gyroscope is essential [2].

Most gyroscope signals disobey superposition and scaling properties, and have a time-varying distribution parameter. In other words, gyroscope signals are usually non-stationary and nonlinear. Many methods for decomposing nonlinear and non-stationary signal, including wavelet transforms (WTs) [3,4,5], empirical mode decomposition (EMD) [6,7,8,9], and variational mode decomposition (VMD) [1,10,11], can be used to denoise gyroscope signals. WTs have very good decomposition ability; however, it is difficult to choose appropriate basis functions and decomposition scales according to specific circumstances. EMD as a widely used method in denoising gyroscope signals needs neither auxiliary function and prior knowledge; however, it has undesirable defects, such as noise-sensitive, mode mixing, and false modes. VMD is proven to outperform EMD in noise robustness and multi-component signal decomposition [11,12]. Therefore, VMD is more suitable for denoising nonlinear and non-stationary signals than EMD. VMD decomposes signal into narrow-band intrinsic mode functions (BLIMFs) with a predefined number of BLIMFs. However, knowing the exact number of BLIMFs for real-life data is always difficult. Recently, a new adaptive data analysis method named adaptive chirp mode pursuit (ACMP) was introduced [13,14]. ACMP captures signal modes one by one in a recursive framework without requiring the number of the signal modes as prior knowledge. Therefore, ACMP is more flexible and easier to operate compared to VMD. By changing the decomposition stopping criterion to mutual information to decide whether or not the decomposition process should stop [15], ACMP is able to extract useful information contained in the gyroscope signal. The signal modes obtained by ACMP still contain noise, more or less according to bandwidth parameter. A larger bandwidth parameter will help the algorithm to find correct modes even when the initial instantaneous frequencies (IFs) are too rough, but it will introduce more noise into the modes. The bandwidth parameter can be set relatively larger to ensure correct modes are obtained, which results in more noise existing in the obtained modes. To improve the denoising effect, signal modes need to be further processed.

There are also many methods for noise reduction without signal decomposition, such as forward linear prediction (FLP) [16,17], moving average (MA) [18], non-local means filter [19], and the Savitzky–Golay (SG) filter [20,21,22]. These methods can be classified as signal smoothing algorithms. FLP models the present sample of a time series as a weighted sum of the past samples. The step-size parameter in FLP algorithm has to be carefully chosen in the case of divergence. Moreover, FLP has poor performance in processing non-stationary signal; it produces a distorted output, especially in sharply changing regions. The moving average filter directly averages a number of points from the input signal, which is very convenient to operate. However, it easily leads to distortion [23]. The non-local means filter denoises the signal by averaging the different regions with similar characteristics. It was originally proposed for image denoising and now has already been used in denoising electrocardiograms [24]. The SG filter is a classic data smoothing method based on local least-squares polynomial approximation. It fits a polynomial to a set of input samples and then evaluates the resulting polynomial at a single point within the approximation interval [22]. The SG filter has no convergence issues and maintains the shape of the signal better in comparison to the moving average filter. Therefore, the SG filter is widely applied for denoising noisy signals. The denoising result depends largely on the window size of the SG filter. The optimal value of the window size has been studied by some scholars [25,26,27]. Although an SG filter with optimal window size will improve the denoising effect to some extent, a fixed-window size SG filter still leads to a trade-off between noise reduction effect and signal preservation. A larger window size suppresses noise more effectively at the expense of distorting signal, especially in dramatically changing regions. To solve this problem, Browne et al. proposed the adaptive window SG filter (AWSGF) [28]. It is based on the idea that white noise is uncorrelated and therefore the Pearson correlation coefficient (PCC) of two subsequent noise sequences is close to zero [29]. However, the noise contained in gyroscope signal is not simple white noise but colored noise. Therefore, the PCC of two subsequent gyroscope noise sequences may sometimes deviate far from zero. Besides, AWSGF’s enormous computation effort greatly limits its use. To solve these problems of AWSGF, we use sample variances based on adaptive moving average (AMA) to adaptively adjust the window size of the SG filter. The AMA technique is used to detect discontinuities in the signal [30]. High sample variance based on AMA indicates the locations of transition [31,32] where the signal should be processed with smaller window size to avoid distortion. Regions with low sample variance should be processed with a larger window size to get satisfying denoising results.

The whole denoising scheme can be concluded as follows. First, the gyroscope signal is decomposed into signal mode one by one using ACMP with stopping criterion based on mutual information. Second, our proposed adaptive multiscale SG filter (AMSGF) is used to further denoise the modes we get in the previous stage. Finally, the denoised signal is obtained as the sum of all processed modes.

This reminder of the paper is organized as follows: Section 2 introduces how to extract useful information contained in the original gyroscope signal by ACMP with modified stopping criterion based on mutual information. Then, AMSGF for further denoising the obtained modes is proposed to improve the performance of the SG filter in Section 3. In Section 4, the denoising results of various real gyroscope experiments are illustrated and compared. Moreover, the denoising effect of AMSGF is presented separately to demonstrate its superiority. Execution time and time complexity are also analyzed in this section. The final conclusions are drawn in Section 5.

## 2. Preliminary denoising based on ACMP

### 2.1. Review of the ACMP Algorithm

Non-stationary signals can be decomposed into several chirp modes, which are modeled as AM-FM signals. The signal model is expressed as the de-chirped form:

(1) $$x=\sum_{m=1}^{M}\alpha_{m}(t)\cos(2\pi \int_{0}^{t}{\tilde{f}}_{m}(s)\mathrm{ds})+\beta_{m}(t)\sin(2\pi \int_{0}^{t}{\tilde{f}}_{m}(s)\mathrm{ds}),$$

with:

(2) $$\alpha_{m}(t)=a_{m}(t)\cos(2\pi \int_{0}^{t}(f_{m}(s)-{\tilde{f}}_{m}(s))\mathrm{ds}+\varphi_{m}),$$

(3) $$\beta_{m}(t)=-a_{m}(t)\sin(2\pi \int_{0}^{t}(f_{m}(s)-{\tilde{f}}_{m}(s))\mathrm{ds}+\varphi_{m}),$$

where $a_{m}(t)$ is the amplitude of the *m*-th mode, $f_{m}(t)$ is the instantaneous frequency (IF), and $\varphi_{m}$ denotes the initial phase. $\alpha_{m}$ and $\beta_{m}$ are two de-chirped signals, ${\tilde{f}}_{m}(t)$ is the frequency function for de-chirping. Based on the idea that the de-chirped signals $\alpha_{m}(t)$ and $\beta_{m}(t)$ will have the narrowest frequency band when $f_{m}(s)$ equals ${\tilde{f}}_{m}(s)$, the optimization problem of the *m*-th signal component can be written as:

(4) $$\underset{\left\{\alpha_{m}\},\{\beta_{m}\},\{{\tilde{f}}_{m}\right\}}{\min}{\left\|\alpha_{m}^{\prime \prime }\right\|}_{2}^{2}+{\left\|\beta_{m}^{\prime \prime }\right\|}_{2}^{2}+\tau {\left\|x(t)-x_{m}(t)\right\|}_{2}^{2},$$

with:

(5) $$x_{m}(t)=a_{m}(t)\cos(2\pi \int_{0}^{t}{\tilde{f}}_{m}(s)\mathrm{ds})+\beta_{m}(t)\sin(2\pi \int_{0}^{t}{\tilde{f}}_{m}(s)\mathrm{ds}),$$

where the first two terms in Equation (4) constrain the two de-chirped signals $\alpha_{m}(t)$ and $\beta_{m}(t)$ to be smooth, the third term represents the energy of the residual signal, τ>0 is the penalty factor. The ACMP greedily finds the desired mode that can take away the most energy from the input signal. The signal is discretized in time $t=t_{0},\ldots ,t_{N-1}$ with *N* samples. Equation (4) can be translated into discrete form as:

(6) $$J_{r}(y_{m},f_{m})={\left\|\Phi y_{m}\right\|}_{2}^{2}+\tau {\left\|x-K_{m}y_{m}\right\|}_{2}^{2},$$

where:

(7) $$x={\left[x(t_{0})\ldots x(t_{N-1})\right]}^{T},\;f_{m}={\left[{\tilde{f}}_{m}(t_{0})\ldots {\tilde{f}}_{m}(t_{N-1})\right]}^{T},y_{m}={\left[\alpha_{m}^{T}\beta_{m}^{T}\right]}^{T},\alpha_{m}={\left[\alpha (t_{0})\ldots \alpha (t_{N-1})\right]}^{T},\;\beta_{m}={\left[\beta (t_{0})\ldots \beta (t_{N-1})\right]}^{T},K_{m}=\left[C_{m}S_{m}\right]$$

with:

(8) $$C_{m}=diag\left[\cos(\theta_{m}(t_{0}))\ldots \cos(\theta_{m}(t_{N-1}))\right],$$

(9) $$S_{m}=diag\left[sin(\theta_{m}(t_{0}))\ldots sin(\theta_{m}(t_{N-1}))\right],$$

where $\theta_{m}\left(t\right)=2\pi \overset{t}{\underset{0}{\int }}{\tilde{f}}_{m}\mathrm{ds}$, $\Phi =\left[\begin{array}{cc} D & 0\\ D & 0 \end{array}\right]$, ***D*** is a second-order difference matrix of size (N−2)×N.

From Equation (6), $y_{m}$ can be updated iteratively using:

(10) $$y_{m}^{n}=\left[\begin{array}{c} \alpha_{m}^{n}\\ \beta_{m}^{n} \end{array}\right]={\left(\frac{1}{\tau }\Phi^{T}\Phi +{\left(K_{m}^{n}\right)}^{T}K_{m}^{n}\right)}^{-1}{\left(K_{m}^{n}\right)}^{T}x.$$

The de-chirped signals are utilized to calculate the IF increment as:

(11) $$\begin{array}{c} \Delta {\tilde{f}}_{m}^{n}\left(t\right)=\;-\frac{1}{2\pi }\frac{d}{dt}\left(\arctan\left(\frac{\beta_{m}^{n}\left(t\right)}{\alpha_{m}^{n}\left(t\right)}\right)\right)=\frac{\beta_{m}^{n}\left(t\right)\cdot {\left(\alpha_{m}^{n}\left(t\right)\right)}^{\prime }-\alpha_{m}^{n}\left(t\right)\cdot {\left(\beta_{m}^{n}\left(t\right)\right)}^{\prime }}{2\pi \left((\alpha_{m}^{n}{\left(t)\right)}^{2}+(\beta_{m}^{n}{\left(t)\right)}^{2}\right)} \end{array}.$$

To prevent the IF from being influenced by noise, the IF increment should be smoothed to satisfy the low-pass property. Finally, the IF increment can be updated as:

(12) $$\Delta f_{m}^{n}={\left(I+\frac{1}{\mu }D^{T}D\right)}^{-1}\Delta {\tilde{f}}_{m}^{n},$$

where μ controls the smooth degree of the IF increment. A smaller *μ* indicates a smoother IF curve.

The IF can be updated as:

(13) $$f_{m}^{n+1}=f_{m}^{n}+\Delta f_{m}^{n}.$$

$\theta_{m}(t)$, $C_{m}$,$S_{m}$, $K_{m}$ can be updated with obtained $f_{m}^{n+1}$, which are used to compute $y_{m}^{n+1}$ by Equation (10). The target signal mode is recovered as:

(14) $$x_{m}^{n}=K_{m}^{n}y_{m}^{n}.$$

The ACMP algorithm can be summarized as follows in Algorithm 1:

**Algorithm 1** ACMP 1: Input signal $x_{o}$; parameters μ>0,τ>0; stopping threshold δ,ε
 2: Set *m* = 1, $r_{1}=x_{o}$
 3: **while**
${\left\|x\right\|}_{2}^{2}/{\left\|x_{o}\right\|}_{2}^{2}>\delta$ do
 4:     Set *n* = 0, obtain the initial IF $f_{m}^{1}(t)$ and $K_{m}^{1}$
 5:     **while**
${\left\|x_{m}^{n}-x_{m}^{n-1}\right\|}_{2}^{2}/{\left\|x_{m}^{n-1}\right\|}_{2}^{2}>\varepsilon$ do
 6:       n=n+1
 7:      compute $y_{m}^{n}$ based on Equation (10)
 8:        compute $x_{m}^{n}$ based on Equation (14)
 9:      update $\Delta f_{m}^{n}$ and $f_{m}^{n+1}$ based on Equation (13)
10:        update kernel matrix $K_{m}^{n+1}$ based on (7), (8), and (9).
11:    **end while**
12:    get signal modes $x_{m}=x_{m}^{n}$
13:    update the residue $x=x-x_{m}$
14:    m=m+1
15: **end while**

The initial instantaneous frequencies can be estimated by detecting the ridge curves of a time-frequency distribution generated by short-time Fourier transform. Useful modes $x_{m}^{n}$ can be extracted one by one from the gyroscope signal. To make sure all useful information is extracted out of gyroscope’s output, we changed the stopping criterion in outer loop (line 3 in Algorithm 1).

### 2.2. Stopping Criterion

**Figure 1:**
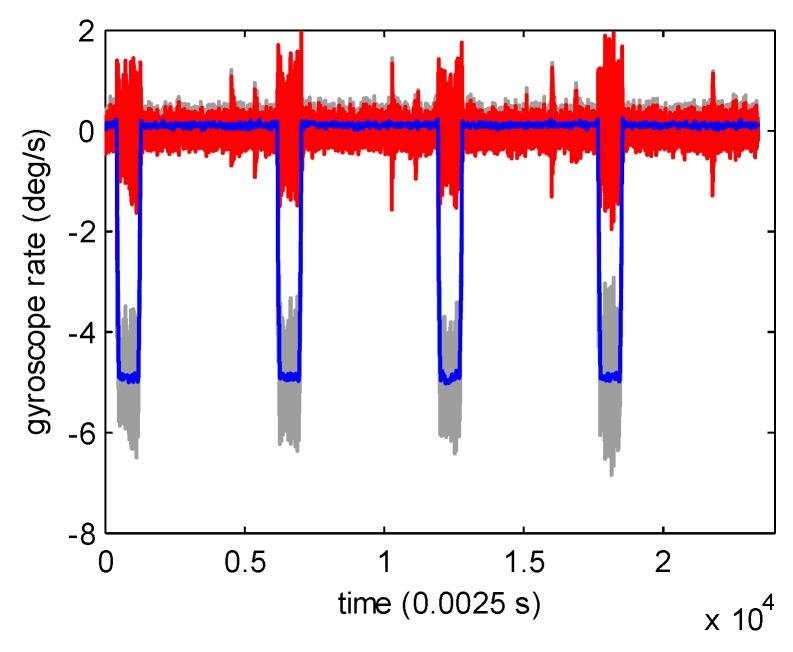
Signal decomposition results (grey: original gyroscope signal; blue: adaptive chirp mode pursuit (ACMP) result; red: residue).

The original stopping criterion in the ACMP algorithm was based on the idea that the decomposition should stop when the ratio of the residue to the original signal is lower than ε. However, the criterion fails in some circumstances. An unsuccessful example is shown in Figure 1. The ratio of the residue to original signal was still up to 0.1527 when the present ACMP result already contained all the useful information of the raw signal. The original stopping criterion was unable to stop the ACMP decomposition duly according to the original stopping criterion, which led to over-decomposition. To avoid this situation, we replaced the original stopping criterion with mutual information. Mutual information measures the mutual dependence between two variables, which is good at identifying correlation degree. Formally, the mutual information of two discrete random variables *X* and *Y* can be defined as:

(15) MI(X,Y)=H(Y)−H(Y|X),

where H(Y) is the entropy of variable *Y*, H(Y|X) is the conditional entropy of variable *Y* when *X* is known. The weaker the correlation between *X* and *Y*, the larger H(Y|X) is. Therefore, MI(X,Y) is small when the correlation between *X* and *Y* is weak. Whether or not the residue contains any important information of original gyroscope signal should become the stopping criterion for ACMP decomposition. We preprocessed the original gyroscope signal using AMSGF, proposed in Section 3. The preprocessing result is denoted as $x_{I}$. $x_{I}$ contains much less noise than the original signal does. Assuming we have already obtained *k* signal modes $x_{i}\left\{i=1,\ldots ,k\right\}$, the residue is expressed as:

(16) $$r_{Ik}=x_{I}-\sum_{i=1}^{k}x_{i}$$

The mutual information between the residue $r_{Ik}$ and $x_{I}$ is $MI(r_{Ik},x_{I})$. We set 0.02 as the decomposition stopping threshold. When $MI(r_{Ik},x_{I})$ is lower than 0.02, we believe the residue rarely contains any useful information of original gyroscope signal. After ACMP, most of the noise is discarded as residue. $MI(r_{Ik},x_{I})$ is 0.0058 in Figure 1, which is lower than decomposition stopping threshold 0.02. By using the proposed stopping criterion, the ACMP decomposition can stop in timely manner.

## 3. Further Denoising Using AMSGF

The bandwidth parameter *τ* in the ACMP algorithm influences the denoising effect. A larger *τ* makes the modes noisier. However, it helps the algorithm decompose the signal into correct modes even if the initial IFs are far from the true IFs. There may be large estimation errors in the initial IFs, especially when the gyroscope signal is complicated. Therefore, a larger τ is preferable ($1e^{-3}$ for all the experiments in this paper), which leads to inevitable noise. The denoising effect is not satisfactory enough, even though the ACMP result has much less noise than the original gyroscope signal, as shown in Figure 1. To reduce the noise contained in the ACMP results, the SG filter was introduced as our further denoising method.

### 3.1. Brief Review of the SG Filter

The SG filter is a classic data smoothing method based on local least-squares polynomial approximation. The signal at sample *x* is smoothed as follows: the *K*-order polynomial function is fitted into the signal in the range of [*x* − *W*, *x* + *W*], where *W* is the predefined window size, and *x* re-indexed as 0 is the center of the group of 2 *W* + 1 input samples:

(17) $$p(x)=\sum_{k=0}^{K}a_{k}x^{k}.$$

The mean-squared approximation error is minimized to obtain the coefficients of the polynomial as:

(18) $$\underset{a_{k}}{\min}\sum_{x=-W}^{W}\sum_{k=0}^{K}{\left(a_{k}x^{k}-f(x)\right)}^{2},$$

where *f*(*x*) is the signal value at sample *x.*

The denoising result of the SG filter depends on the predefined polynomial degree *K* and window size 2 *W* + 1. The choice of the window size has to be particularly considered to avoid distortion of the signal. A non-stationary signal always entails both sharply changing regions and flat regions. A small window size should be applied to the former to ensure less distortion, while a large window size ensures less noise in flat regions. In that case, a fixed window size is unable to balance a smaller bias error with less noise. Based on the idea that the window size should be able to change adaptively in different kinds of regions, AMA was used to adjust the window size.

### 3.2. Sample Variance Based on AMA

The AMA technique is used to detect discontinuities in signal. It adjusts the window size of the moving average according to the rate of change of the signal [30].

AMA is expressed as follows:

(19) $$Y_{t}=\frac{1}{q_{H}+q_{L}}\sum_{i=-q_{T}(t)}^{-q_{H}(t)}X_{t+i},$$

where

(20) $$q_{H}(t)=\left\{ \begin{array}{cc} q & if\;D^{\prime }(t)\geq 0\\ f(D(t))\cdot q & if\;D^{\prime }(t)<0 \end{array}\right.,$$

(21) $$q_{L}(t)=\left\{ \begin{array}{cc} q & if\;D^{\prime }(t)>0\\ f(D(t))\cdot q & if\;D^{\prime }(t)\leq 0 \end{array}\right.,$$

(22) $$f(D(t))=1-\frac{D(t)}{\max(D(t))},$$

(23) D(t)=|y(t+q)−y(t−q)|,

(24) $$D^{\prime }(t)=D(t+1)-D(t).$$

The moving average process is repeated iteratively for *p* times, with $Y_{t}$ replacing $X_{t}$ in Equation (19) to get the final moving average result. The sample variance is finally obtained by:

(25) $${\hat{\sigma }}_{t}^{2}=\frac{\sum_{i=q_{L}}^{q_{H}}{\left(Y_{i}-{\bar{Y}}_{t}\right)}^{2}}{q_{H}+q_{L}}.$$

**Figure 2:**
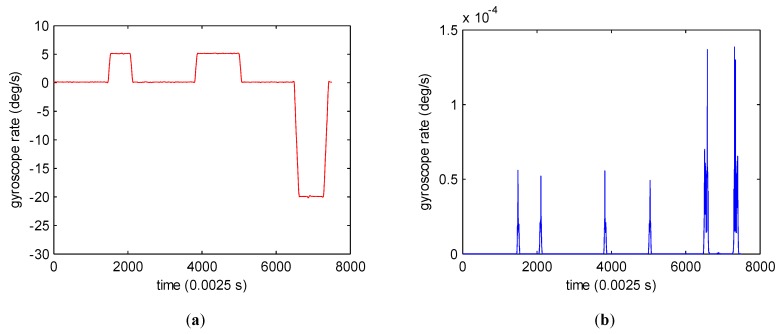
Sample variance. (**a**) Gyroscope signal. (**b**) Sample variance.

High sample variance means dramatically changing position in signal. For example, the gyroscope signal changes sharply at sample 1461, 2133, 3815, 4956, 6476, and 7431, as shown in Figure 2a, and the sample variance is high at the same position accordingly, as shown in Figure 2b.

### 3.3. AMSGF Based on AMA

High sample variance indicates the existence of a dramatic change in signal, while low sample variance relates to smoothness. Therefore, sample variance can help find the transition of the signal and adjust the window size of the SG filter for each sample adaptively. Large window size should be selected for high sample variance regions to get a better denoising effect and small window size for low sample variance regions to avoid signal distortion. If sample variance ${\hat{\sigma }}_{t}^{2}>\lambda_{th}$, we assumed there was sudden change and decreased the window size correspondingly, where $\lambda_{th}$ is the sample variance threshold to detect transition in gyroscope signal. We denoted samples with sample variance higher than $\lambda_{th}$ as $x_{h}$, samples with sample variance lower than $\lambda_{th}$ as $x_{l}$. The proposed AMSGF can be depicted as:

Step 1: Choosing $W_{\max}$, $W_{\min}$, $\lambda_{th}$, *M* as the initial value. $2W_{\max}+1$ is the maximal window size; $2W_{\min}+1$ is the minimal window size. *M* is the order of polynomial function in Equation (17);

Step 2: Calculating the sample variance $V(x_{i})$ of each sample $x_{i}$ from Equations (19)–(25);

Step 3: Adjusting the window size of SG filter at each sample $x_{i}$ according to $V\left(x_{i}\right)$ using:

(26) $$W_{L}(x_{i})=\left\{ \begin{array}{cc} W_{\max} & V(x_{i-l})<\lambda_{th},\;l=0,\ldots ,W_{\max}\\ \max(l,W_{\min}) & V(x_{i-l})<\lambda_{th},\;V(x_{i-l-1})>\lambda_{th},\;0\leq l<W_{\max}-1 \end{array}\right.,$$

(27) $$W_{R}(x_{i})=\left\{ \begin{array}{cc} W_{\max} & V(x_{i+l})<\lambda_{th},\;l=0,\ldots ,W_{\max}\\ \max(l,W_{\min}) & V(x_{i+l})<\lambda_{th},\;V(x_{i+l+1})>\lambda_{th},\;0\leq l<W_{\max}-1 \end{array}\right.,$$

where $W_{L}$ is the left half window size of the SG filter at sample $x_{i}$; $W_{R}$ is the right half window size of SG filter at sample $x_{i}$. $W_{L}+W_{R}+1$ the total window size at sample $x_{i}$;

Step 4: Calculate the smoothed result at $x_{i}$ using Equations (17) and (18). The *K*-order polynomial function is fitted into the signal in the range of $\left[x_{i}-W_{L}(x_{i}),\;x_{i}+W_{R}(x_{i})\right]$.

As Equations (26) and (27) show, samples considered to be at smooth regions were processed with a large window size so that noise could be eliminated to the greatest extent. Meanwhile, samples considered to be at dramatically changing regions were processed with a small window size to prevent signal distortion. The window size was adjusted between $2W_{\min}+1$ and $2W_{\max}+1$ adaptively according to the value of sample variance.

**Figure 3:**
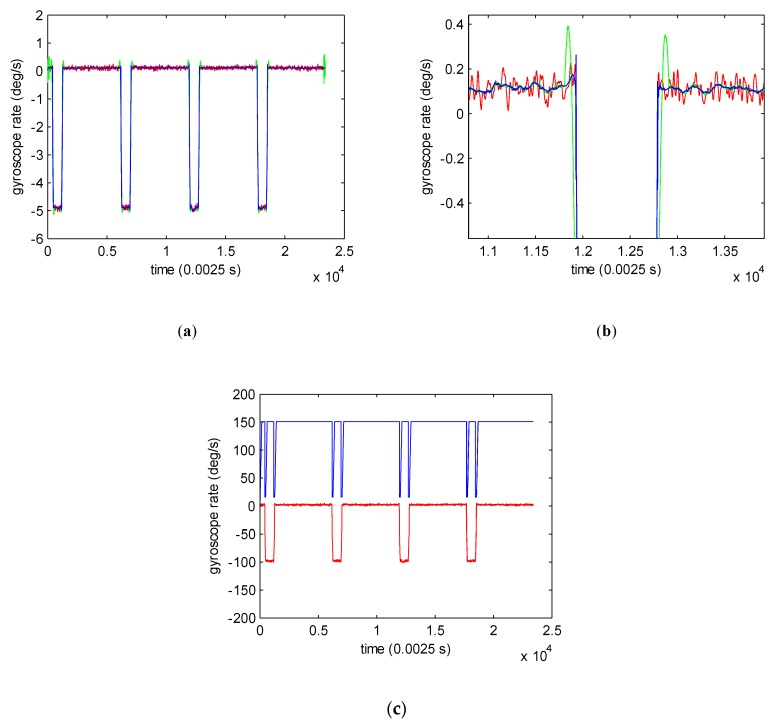
Denoising results of the adaptive multiscale SG filter (AMSGF) and fixed-window-size Savitzky–Golay (SG) filter. (**a**) Denoising results using AMSGF, $W_{\max}=151,W_{\min}=15,\;\lambda_{th}=2.5\times 10^{-6},\;M=3.$ (Red: ACMP results of the original gyroscope signal; blue: further denoising results using AMSGF; green: further denoising results using the fixed window size SG filter with fixed window size of 151). (**b**) Local magnified image of denoising results in (**a**). (**c**) Window size selection of AMSGF at different samples (red: 500 times magnified ACMP results of the original gyroscope signal. blue: window size selection results at different sample).

We compared AMSGF with a fixed window size SG filter to test its improvement. The denoising results of different methods are shown in Figure 3a. As we can see from Figure 3b that there are obvious differences in the transition of the signal around $1.19\times 10^{4}$ and $1.28\times 10^{4}$. The fixed window size SG filter produced serious distortion in dramatically changing regions when obtaining the same denoising effect as AMSGF, while AMSGF resulted in small distortion without the compromising good denoising effect. The window size selection results based on sample variance for AMSGF are shown in Figure 3c. The window size declined sharply near the transition of signal. In conclusion, compared to the fixed window size SG filter, AMSGF can get both good noise-reduction effect and signal preservation effect.

**Figure 4:**
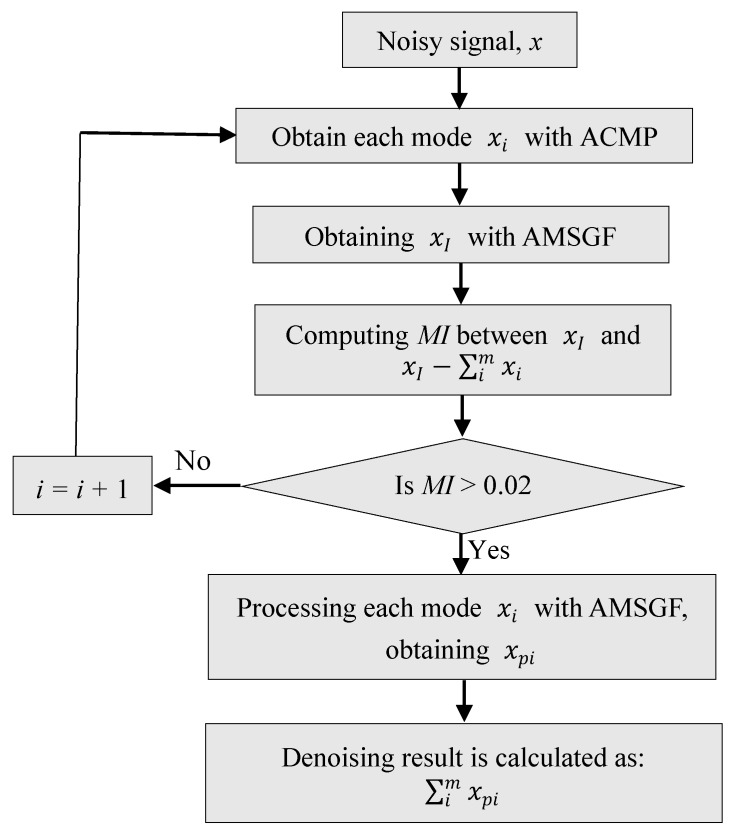
Flow chart for the proposed algorithm.

A flow chart for the proposed algorithm is summarized as depicted in Figure 4. First, the original gyroscope signal is processed with ACMP to obtain modes that mainly contain useful information. As the signal is decomposed into modes one by one, the mutual information is calculated to decide whether or not the decomposition should stop. After ACMP, most noise is discarded in the residue. However, there is still more or less unexpected noise remaining in the modes. Then, each mode is denoised further using AMSGF. The window size is chosen adaptively according to the sample variance based on AMA for each sample. Finally, all denoised modes are summed up to constitute the denoising gyroscope signal.

## 4. Results and Discussion

**Figure 5:**
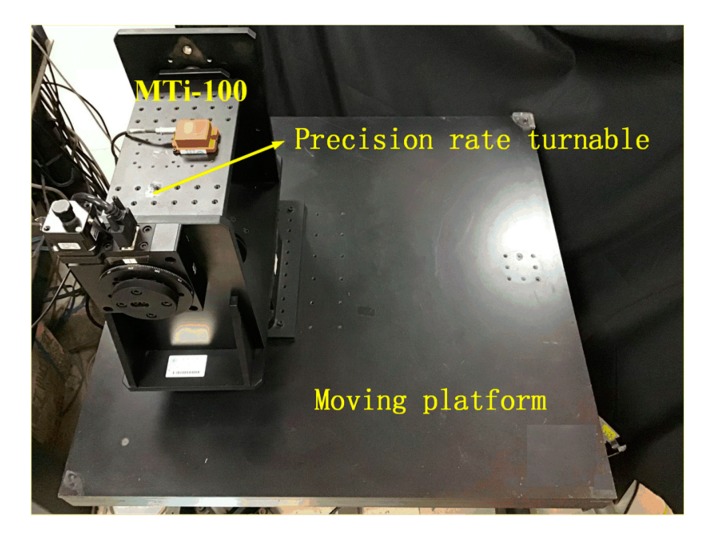
Experimental setup.

In this section, practical gyroscope rate experiments in different motion states are performed to verify the effectiveness of the proposed denoising method. The experimental setup consisted of a triaxial micro-electromechanical system (MEMS) gyroscope MTi-100 made by Xsens Technologies (Ernshard, Holland), precision rate turntable (Zolix, Beijing, China), and moving platform (AllController, Nanjing, China), as shown in Figure 5. The gyroscope rate data were collected in a static state, regular multiple-rate state, and arbitrary multiple-rate. We compared the denoising results of our proposed algorithm with those of the empirical mode decomposition soft interval thresholding (EMD-SIT) denoising method, wavelet threshold denoising method, and VMD-based denoising, which are the most widely used methods for off-line denoising. Here, we used the consecutive mean square errors (CMSE) proposed in [33] to determine the M1 and similarity measure between the probability density functions of intrinsic mode functions (IMFs) proposed in [34,35] to determine M2 for choosing EMD-SIT parameters [36]. The wavelet threshold denoising method used as a comparative method was described in [37] with noise variance estimation based on Gaussian mixture model classification. VMD-based denoising was described in [11], which combined VMD with the detrended fluctuation analysis.

Moreover, AMSGF was compared with common smoothing methods separately to illustrate its superiority in denoising a non-stationary gyroscope signal. Comparative methods included FLP, AWSGF, and non-local means filter. Here, we chose the step-size of FLP and the half width of non-local means to achieve their best denoising results with acceptable signal distortion.

### 4.1. Static Motion Experiments

**Figure 6:**
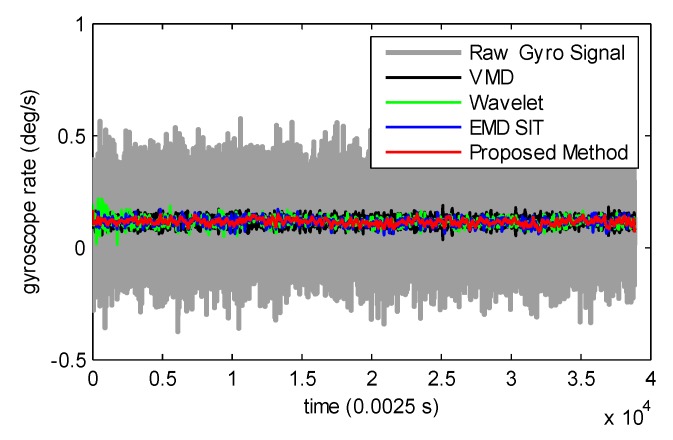
Denoising results of different methods for static motion experiments.

The denoising results of different methods for static gyroscope data are shown in Figure 6. The corresponding signal to noise ratio (SNR) results are listed in Table 1. Our proposed method was shown to acquire the optimal results as compared to the reference algorithms. The SNR increased from −0.47 dB to 19.3 dB after the original gyroscope signal being denoised by our proposed method, which testifies that our proposed method can effectively reduce the random error contained in gyroscope signals.

### 4.2. Regular Multiple-Rate Motion Experiments

**Figure 7:**
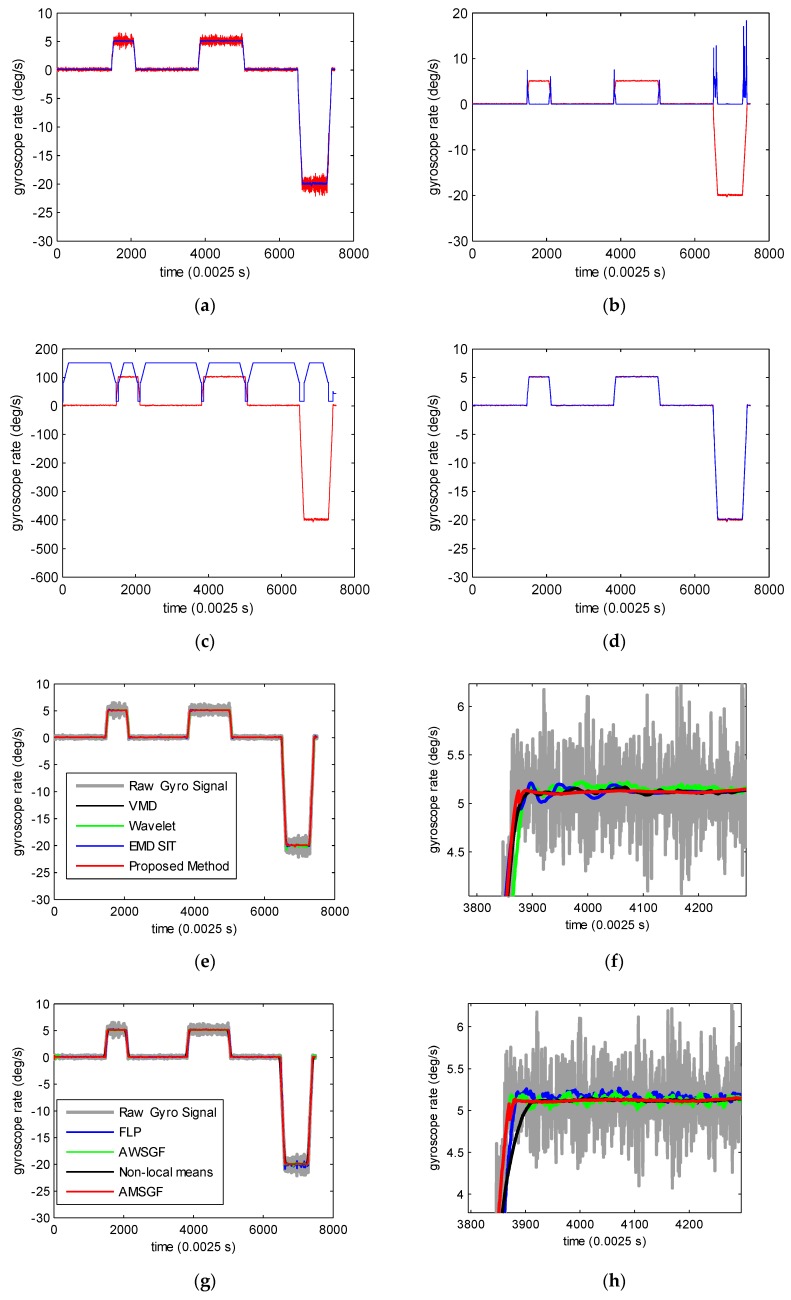
Regular multiple-rate motion experiments. (**a**) ACMP results (red: original gyroscope signal; blue: ACMP results). (**b**) Sample variance (red: ACMP results; blue: 50,000 times magnified sample variance). (**c**) The window size for different samples, $W_{max}=151,\;W_{min}=15,\;\lambda_{th}=2.5\times 10^{-6},\;M=3\;$ for AMSGF (red: 10 times magnified ACMP results; blue: window size selection of AMSGF for different samples). (**d**) Final denoising results (red: ACMP results; blue: further denoising results using AMSGF). (**e**) Denoising results of different methods. (**f**) Local magnified image of denoising results in (**e**). (**g**) Denoising results of different smoothing methods. (**h**) Local magnified image of denoising results in (**g**).

The precision rate turntable moved at a piecewise constant rate with the gyroscope connected to it. The denoising results are shown in Figure 7. As we can see from Figure 7a, the signal was less noisy after being processed by ACMP. The characteristics of the sample variances of regular multiple rate motion were obvious, especially the values of sample variances with rate change, which were large enough, as depicted in Figure 7b. Therefore, the window size was small at the moments of rate change and large at flat regions, as shown in Figure 7c. Further denoising results obtained by AMSGF are shown in Figure 7d.

From Figure 7e and its local enlarged image Figure 7f, it can be easily seen that our proposed algorithm can effectively reduce the noise in a gyroscope signal. Our proposed method clearly obtains the best denoising results in the smooth region without distortion in signal transition because of the adjustment of AMSGF’s window size. Figure 7g and its local enlarged image Figure 7h prove that AMSGF performed better than FLP and AWSGF in flat regions, and the non-local means filter in changing regions. The signal processed by FLP still had a lot of unexpected fluctuations. The non-local means filter had a much better performance in the smooth region than the sharply changing region. AWSGF can get good denoising results when the gyroscope is static. However, its denoising effect degrades when the gyroscope moves. The main reason is that noise contained in static gyroscope output is uncorrelated, so it satisfies the AWSGF’s assumption. However, noise contained in the moving gyroscope’s output cannot satisfy the uncorrelated assumption. The SNR results of signal denoised by different methods are listed in Table 2 and Table 3, which illustrate that our proposed method gained the best denoising effect among all these methods and AMSGF can get better SNR results than comparative smoothing methods.

### 4.3. Arbitrary Multiple-Rate Motion Experiments

**Figure 8:**
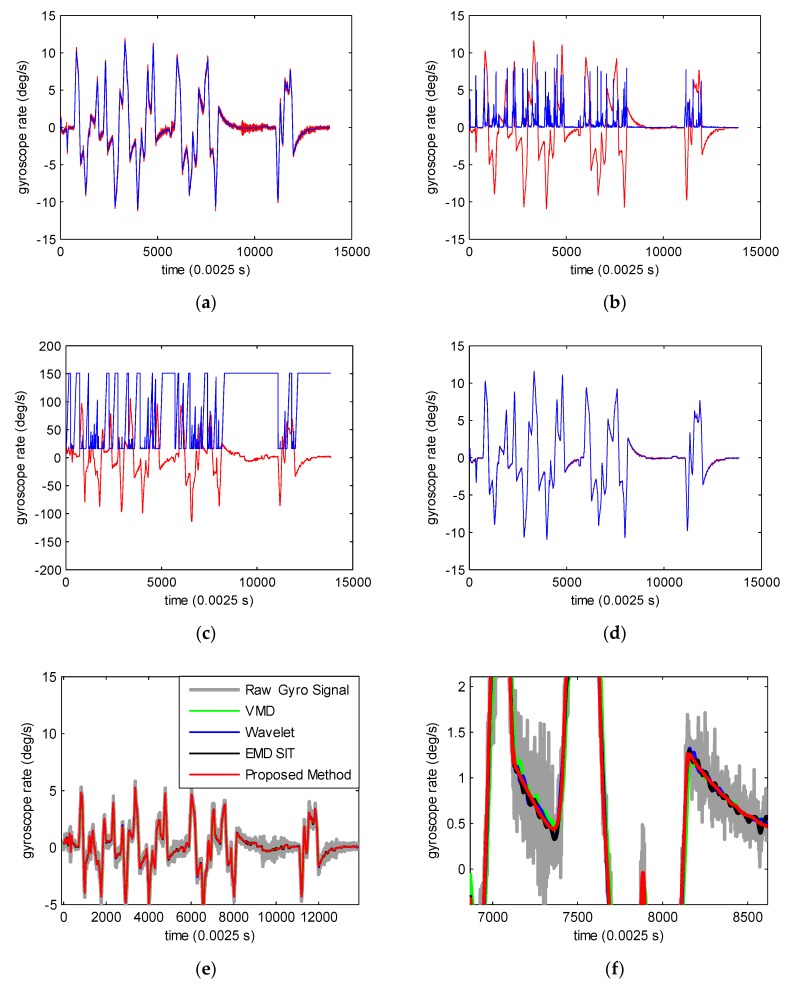

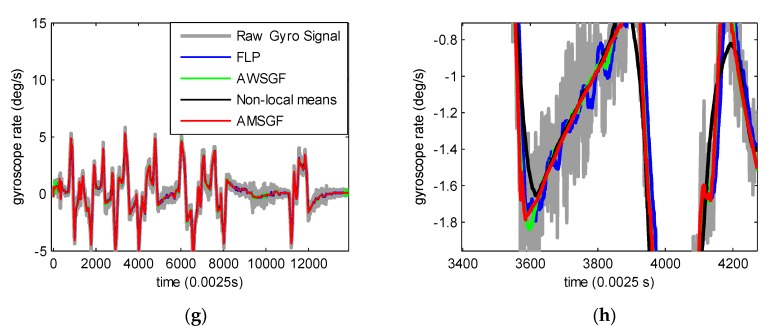
Arbitrary multiple-rate motion experiments. (**a**) ACMP results (red: original gyroscope signal; blue: ACMP results). (**b**) Sample variance (red: ACMP results. blue: 50,000 times magnified sample variance). (**c**) The window size for different samples, $W_{max}=151,\;W_{min}=15,\;\lambda_{th}=2.5\times 10^{-6}\;,\;M=3\;$ for AMSGF (red: 10 times magnified ACMP results; blue: window size selection of AMSGF for different samples). (**d**) Final denoising results (red: ACMP results. blue: further denoising results using AMSGF). (**e**) Denoising results of different denoising methods. (**f**) Local magnified image of denoising results in (**e**). (**g**) Denoising results of different smoothing methods. (**h**) Local magnified image of denoising results in (**g**).

The gyroscope fixed to the precision rate turntable was placed upon the moving platform. The platform moved randomly to generate an arbitrary multiple-rate gyroscope signal. As shown in Figure 8a, the noise contained in the gyroscope signal was greatly reduced after being processed by ACMP. Figure 8b depicts the sample variance, which was evidently lower in smoothly changing regions. Different window size selection for different samples for AMSGF is shown in Figure 8c. As we can see, a small window size was used at sharply changing regions to avoid distortion, while a large window size was selected for smoothly changing regions for noise reduction. Figure 8d displays further denoising effects of AMSGF. The denoising results of different methods are shown in Figure 8e. The local magnified image of denoising results in Figure 8f shows the effectiveness of the proposed methods in denoising the gyroscope signal. From Figure 8f, it can be seen that the proposed method led to a better denoising effect, especially when the rate changed slowly. The reason is that AMSGF uses a longer window size instead of the fixed window size used by other smoothing methods to smooth gyroscope data to eliminate the noise when the rates change slowly. The denoising results in Figure 8g,h also illustrate this point.

### 4.4. Execution Time

For each *n*-length signal, we took the mean of ten tests as our final execution time result. All tests were run on matlab 2014. The execution time is shown in Table 4. The time complexity of the proposed method is O(*n*). The proposed method was more time-consuming than Wavelet and EMD-SIT, but it was much more time-efficient than VMD-based denoising. The proposed method combined ACMP with AMSGF. The time spent on AMSGF took up a great proportion of the whole execution time. For $n=2^{14}$, getting the initial value of ACMP consumed 0.53 s; signal decomposition using ACMP consumed only 0.12 s. AMSGF was much slower than FLP and a bit slower than non-local means in our tests. The reason is that AMSGF needs to calculate the AMA of each point in advance and find the window size of each point based on AMA while smoothing the signal. AMSGF was much faster than AWSGF, as we expected. AWSGF needs extremely inefficient computational implementation because it smooths the data gradually, increasing filter length.

## 5. Conclusions

In this paper, a hybrid method combining ACMP with an adaptive multiscale SG filter was proposed. The proposed method is suitable for denoising off-line non-stationary gyroscope rate signals. ACMP decomposes the signal into signal modes one by one with the mutual information used to determine whether or not the decomposition should stop. Most of the noise contained in the gyroscope signal is discarded as decomposition residue. We applied an improved SG filter, a further smoothing method, to denoise the signal modes. The fixed window size SG filter usually has poor performance in balancing noise reduction with signal preservation. To overcome this drawback of the SG filter, we introduced sample variance based on AMA to adjust the window size of the SG filter adaptively at different signal samples. AMSGF was proposed by modelling the sample variance with the window size.

Practical data collected from MEMS gyroscope were used to evaluate the performance of the proposed method. The experiments of different motion forms (single rate motion, regular multiple rates motion, and arbitrary multiple rates motions) demonstrated that the proposed method is suitable for off-line signal noise reduction, not only in a static state or low dynamic state but also a highly dynamic state. Besides, AMSGF was proven to be superior to the reference smoothing methods. Further work needs to concentrate on more efficient computational implementation, optimal selection of the maximum window size, minimum window size, sample variance threshold, and the filter order in AMSGF. Moreover, this paper only considered gyroscope signal denoising. Experiments will be done to test the proposed method’s denoising effect with other types of signals in the future.

## Figures and Tables

**Table 1:** Results of the micro-electromechanical system (MEMS) gyro signal in static motion experiments.

	Raw Signal	Proposed Method	VMD	Wavelet	EMD SIT
SNR (dB)	−0.47	19.37	14.31	16.72	16.51

**Table 2:** SNR comparison with classic off-line denoising methods.

	Raw Signal	Proposed Method	VMD	EMD SIT	Wavelet
SNR (dB)	26.58	52.91	47.74	42.82	46.24

**Table 3:** SNR results of different smoothing methods.

	Raw Signal	AMSGF	FLP	AWSGF	Non-local Means
SNR (dB)	26.52	51.04	40.20	44.22	45.09

**Table 4:** Execution time (s).

*n*	Proposed Method	Wavelet	VMD	EMD SIT	AMSGF	FLP	AWSGF	Non-Local Means
$2^{9}$	0.21	0.064	1.22	0.18	0.18	0.0048	11.21	0.16
$2^{10}$	0.38	0.064	2.30	0.20	0.31	0.0063	30.43	0.29
$2^{11}$	0.68	0.065	5.67	0.28	0.59	0.012	67.21	0.51
$2^{12}$	1.35	0.064	14.08	0.55	1.14	0.026	176.25	1.01
$2^{13}$	2.61	0.079	27.17	0.74	2.25	0.044	467.24	1.82
$2^{14}$	5.22	0.083	65.57	2.30	4.47	0.13	1465.66	3.81
